# *Leptospira* spp. Prevalence in Cats from Southern Italy with Evaluation of Risk Factors for Exposure and Clinical Findings in Infected Cats

**DOI:** 10.3390/pathogens11101129

**Published:** 2022-09-30

**Authors:** Giulia Donato, Marisa Masucci, Katrin Hartmann, Marga G. A. Goris, Ahmed A. Ahmed, Joy Archer, Angela Alibrandi, Maria Grazia Pennisi

**Affiliations:** 1 Department of Veterinary Sciences, University of Messina, 98168 Messina, Italy; 2 Medizinische Kleintierklinik, LMU Munich, 80539 Munich, Germany; 3 Department of Medical Microbiology, OIE and National Collaborating Centre for Reference and Research on Leptospirosis, Amsterdam University Medical Center, University of Amsterdam, 1105 Amsterdam, The Netherlands; 4 Department of Veterinary Medicine, University of Cambridge, Madingley Road, Cambridge CB3 0ES, UK; 5 Unit of Statistical and Mathematical Sciences, Department of Economics, University of Messina, 98122 Messina, Italy

**Keywords:** leptospirosis, leptospires, infection, zoonosis, feline, microscopic agglutination test, MAT, polymerase chain reaction, PCR, epidemiology

## Abstract

Leptospirosis is a worldwide zoonotic disease, but feline leptospirosis is rarely reported. This study aimed at investigating *Leptospira* spp. prevalence in cats from southern Italy, evaluating risk factors, clinical findings and laboratory data associated with infection. The serum of 112 cats was investigated by microscopic agglutination test (MAT), detecting anti-*Leptospira* antibodies against 14 pathogenic serovars. Blood and urine samples were tested by a real-time polymerase chain reaction targeting the *lipL32* gene of pathogenic *Leptospira*. Antibodies against serovars Poi, Bratislava, Arborea, Ballum, Pomona and Lora were detected in 15.3% (17/111) of cats (titers range: 20–320). *Leptospira* spp. DNA was found in 3% (4/109) of blood and 9% (10/111) of urine samples. The spring season was the only risk factor for urinary *Leptospira* DNA shedding. Laboratory abnormalities significantly associated and/or correlated with *Leptospira* spp. positivity were anemia, monocytosis, neutrophilia, eosinopenia, increased alanine aminotransferase activity, hypoalbuminemia and hyperglobulinemia. In the investigated areas, cats are frequently infected by *Leptospira* spp. and can represent an additional reservoir or sentinel for a risk of infection. Moreover, some laboratory changes could be compatible with a pathogenic effect of *Leptospira* spp. in the feline host.

## 1. Introduction

Leptospirosis is a zoonotic disease caused by Gram-negative, spirochetal bacteria belonging to the genus *Leptospira*. Leptospires are highly motile, elongated, helically coiled bacteria characterized by hook-shaped ends [1,2,3]. Currently, *Leptospira interrogans* sensu latu includes more than 260 pathogenic serovars belonging to 26 serogroups [1], and according to the European consensus statement on leptospirosis in dogs and cats, serogroups Icterohaemorragiae, Canicola, Grippotyphosa, Pomona, Sejroe, Ballum, Autumnalis and Bratislava are the most frequently identified in cats [2]. Leptospirosis is reported worldwide, and cases of feline leptospirosis have been described in Africa (Algeria and Reunion Island) [4,5,6], Asia (Iran, Japan, Malaysia, Taiwan and Thailand) [7,8,9,10,11,12,13,14], America (Brazil, Chile, Canada, Mexico, the Caribbean island of Saint Kitts and the USA) [15,16,17,18,19,20,21,22,23,24,25,26], Europe (the Czech Republic, Estonia, Germany, Greece, Serbia and Spain) [27,28,29,30,31,32,33], the United Kingdom (Scotland) [34], and Oceania (New Zealand and Australia) [35,36,37]. In Italy, Stefanetti and others [38] retrospectively investigated the role of *Leptospira* spp. in cases of abortion, stillbirth and neonatal mortality in cats, but none of the pathologic samples (placenta, pooled organs of fetuses and neonates) were found positive. Antibody prevalence and prevalence of *Leptospira* DNA in urine reported in cats range between 4% to 33.3% and 0% to 67.8%, respectively [1]. Although clinical signs in infected cats seem to be rare, they can shed leptospires in their urine and might represent a reservoir or incidental hosts in the transmission [1,2,3]. The close interaction between cats and humans offers ideal conditions for zoonotic transmission [39]. Cats can become infected by hunting prey that harbors leptospires or after exposure to infectious urine of cohabiting dogs, cats, and other species like pigs and cows [1,2,3]. Living outdoors, in urban areas and being an old cat or a hunting cat have been reported as possible risk factors for *Leptospira* spp. exposure. No associations have been reported with sex and/or breed [2,3]. Although in Italy, cases of leptospirosis are widely reported in dogs [40,41,42,43,44,45,46] and humans [47,48,49,50] and *Leptospira* spp. infection has been reported in other host species [51,52,53,54,55,56,57,58,59,60,61,62], only one study so far has investigated *Leptospira* spp. prevalence in cats [38]. The present study aimed to investigate *Leptospira* spp. prevalence in cats from southern Italy, evaluating risk factors for exposure and describing clinical findings and laboratory data on infected cats.

## 2. Results

### 2.1. Cat Demographic, Clinical and Clinicopathological Data

One hundred and twelve cats were included in the study, of which 111 blood serum samples, 111 urine samples and 109 K_3_EDTA blood samples were collected. 

Twenty-six cats (23.2%) were from Sicily and 86 cats (76.8%) were from Calabria. Cats coming from Sicily and Calabria differed in age, lifestyle and environment. Particularly, adult cats (Fisher’s exact test, *p* = 0.0284) and cats living in suburban areas (Fisher’s exact test, *p* = 0.0354) were more frequently enrolled in Sicily, and most of the cats from Calabria lived outdoors rather than indoors (Fisher’s exact test, *p* = 0.0354) or indoor/outdoor (Fisher’s exact test, *p* = 0.0196). 

Signalment and history of the enrolled cats are shown in Table 1, and the clinical findings are reported in Table 2. Clinicopathological abnormalities are reported in the supplementary Table S1. Cats were aged between 5 and 204 months (median 24 months, 25th percentile 10.5 months and 75th percentile 72 months). At physical examination, nasal discharge was reported as the only respiratory tract sign; gastrointestinal signs included stomatitis, vomiting and diarrhea; skin lesions were alopecia, hyperkeratosis, scaling, and abscess; and ocular signs included conjunctivitis and keratoconjunctivitis. 

### 2.2. Leptospira spp. Antibody Prevalence

Antibodies against *Leptospira* spp. were detected in 15.3% (17/111) of the cats. Antibody titers ranged from 20 to 320. The most frequently detected positivity was against serovar Poi (9/17 of Ab^+^ cats), followed by Bratislava (5/17) and Arborea (3/17), while antibodies against serovars Ballum, Pomona, Lora and Mini were less frequently detected (Table 3). Antibody titers against at least two serovars belonging to different serogroups were detected in two cats: one cat (cat 2) was positive for Jez Bratislava (titer 20) and Poi (titer 20) strains belonging to different serogroups; another cat (cat 6) was positive for the Jez Bratislava (titer 320) and Lora (titer 80) strains belonging to the same serogroup (Australis), and for the Sari (titer 80) and Arborea (titer 40) strains belonging to two other serogroups (Table 3). Four Ab^+^ cats (cats 6,8,9,16) shed pathogenic *Leptospira* DNA in their urine, and in two other cats (cats 3, 7), leptospiraemia was observed (Table 3).

### 2.3. Leptospira spp. DNA Detection in Blood and Urine

Three percent (4/109) of K_3_EDTA blood samples and 9% (10/111) of urine samples evaluated by polymerase chain reaction (PCR) were positive, with an overall molecular prevalence of 12% (14/112). No cats were simultaneously positive in blood and urine. 

Twenty-five of the 111 cats were positive for at least one test, with an overall prevalence (Ab^+^/ DNA^+^) of 22.5%. A positive correlation between antibody positivity and urinary DNA shedding (Spearman’s Rho test, r_s_ = 0.215; *p* = 0.024) was found.

Based on PCR and antibody data, four different patterns were evidenced, and they are reported in Table 4 together with their significant associations.

### 2.4. Risk Factors and Correlation with Clinical and Laboratory Variables

The spring season and *Leptospira* DNA detection in urine were significantly associated (univariate logistic regression analysis: *p* = 0.032, OR = 4.327, 95% CI = 1.131–16.549; multivariate logistic regression analysis: *p* = 0.034, OR = 4.871, 95% CI = 1.127–21.057). Moreover, urinary *Leptospira* DNA shedding was more frequently found in cats enrolled during the spring season compared to those enrolled during wintertime (Fisher’s exact test; *p* = 0.0184, OR = 6.808, 95% CI = 1.531–34.15). None of the three cats enrolled in the summer tested positive. 

Statistically significant haematological and biochemical changes according to *Leptospira* spp. DNA and antibody positivity are shown in Table 5. *Leptospira* spp. antibody positivity was significantly associated (Fisher’s exact test) with monocytosis and positively correlated (Spearman’s Rho test) to monocyte count, anemia and increased ALT values. *Leptospira* spp. DNA detection was positively correlated to monocyte (uDNA^+^; DNA^+^) and neutrophil (bDNA^+^; DNA^+^) counts (Spearman’s Rho test) and significantly associated (Fisher’s exact test) with hyperglobulinemia (uDNA^+^) and neutrophilia (DNA^+^). Anemia, neutrophilia, monocytosis and hypoalbuminemia were significantly associated (Fisher’s exact test) and positively correlated (Spearman’s Rho test) with *Leptospira* positivity in at least one test (Ab^+^/ DNA^+^). Anemia, neutrophilia and monocytosis were also significantly associated (Fisher’s exact test) and positively correlated (Spearman’s Rho test) with *Leptospira* Ab^+^/uDNA^+^. Eosinopenia was significantly associated (Fisher’s exact test) with *Leptospira* Ab^+^/uDNA^+^ (Table 5). No significant associations were found with the type of anemia and reticulocyte hemoglobin content (RETIC-HGB).

## 3. Discussion

This is the first study that reported the prevalence of *Leptospira* spp. in a population of cats from Southern Italy (Sicily and Calabria regions), evaluating antibody prevalence and the presence of DNA in blood and urine. Considering the differences existing in the feline population of the Sicily and Calabria regions, the prevalence of *Leptospira* spp. in the two regions was not compared. Antibodies against *Leptospira* spp. were detected in 15.3% of the cats, with titers ranging from 20 to 320. The cut-off dilution used in the present study for positivity was 1:20, in accordance with the one used in previous epidemiological studies [8,33], but lower than in most other studies where 1:100 was considered the cut-off dilution [1]. However, in the present study, antibody detection was aimed at investigating the exposure of studied cats to *Leptospira* spp. at some point in their lives, and the level of positivity of samples was not relevant. Therefore, a dilution of 1:20 was considered appropriate, as reported previously [8,33]. Moreover, there is no consensus on the most appropriate cut-off value in cats, and cats seem to respond to infection with low antibody titers [8,22,26,29,34,35]. The reason for low titers in cats tested in this study could be due to serovars not tested and/or cross-reaction with some others that we tested. Moreover, infected cats might mount a lower antibody response compared to dogs [8,19]. Additionally, cats generally could have a short-term immune response, with a rapid decline in titers [22,35]. 

The most common serovar detected in the study was Poi (9/17 of antibody positive cats), followed by Bratislava (5/17 of antibody positive cats) and Arborea (3/17 of antibody positive cats), and less frequently, serovars Ballum, Pomona, Lora and Mini were found. Unexpectedly, positivity for serogroups reported to be the most frequently involved in feline leptospirosis in Europe, according to the European consensus statement on leptospirosis, such as Canicola, Grippotyphosa, Sejroe and Icterohaemorrhagiae [1,2], was not detected. Antibodies against the serovars Poi, Arborea, and Mini were never reported before in cats; however, the most frequent seroreactivity found in the present study involved serovars previously described in other hosts in Italy, such as wild boars [59], pigs [60,61], wolves [62], Hystrix cristata [56], horses [52,55], dairy cattle [54] and wild ruminants [51], kennel dogs [42,43,46] and humans [50]. Moreover, Tagliabue et al. [43] reported in various host species, antibody-positivity against serogroups Australis (dogs, wild boars, horses, hares, swine, foxes and rodents), Sejroe (cattle, sheep, goats and buffaloes), Icterohaemorrhagiae (dogs, goats and foxes), Pomona (swine, cattle and wild species) and Grippotyphosa (hares). 

In the present study, 3% of blood samples and 9% of urine samples were PCR positive. Few have studies investigated *Leptospira* spp. in blood samples, reporting a prevalence ranging from 1.12% to 11.9% [1,7,13,65]. Instead, a few more studies have investigated *Leptospira* spp. DNA shedding in urine [7,8,14,19,26,27,30,33,65] reporting a prevalence ranging from 0% to 67.8% [1], with the possibility of long-lasting leptospiral DNA shedding (eight months) after the first presentation [27]. According to the European consensus statement on leptospirosis [2], the MAT is the most widely used diagnostic test for acute leptospirosis in dogs; however, it does not provide any information about whether an animal is a carrier. Cats are supposed to respond to infection with a rapid immune response, followed by a rapid decline in titers [22,35] and high titers can reflect either a recent or active infection, or re-infection [19]. In dogs with consistent clinical signs, a positive blood PCR is suggestive of acute leptospirosis, while a positive urine PCR indicates renal shedding, which can occur in both acutely infected animals and chronic renal carriers [2]. In dogs, leptospiraemia can be found for the first 10 days after infection and thereafter, leptospires can be found in urine. However, leptospiraemia is transient and urinary shedding can be intermittent, and therefore, a negative result in these samples does not rule out leptospirosis [2]. In two older experimental studies, cats were infected orally [66] or subcutaneously [67], and leptospires were detected by blood and urine bacterial culture. Leptospiraemia was observed [66] 6–10 days post infection (p.i.) and persisted for 1–7 days. Leptospiruria was documented in both studies after 12–28 days p.i. and persisted for 2–8 weeks [66,67]. However, anti-*Leptospira* spp. antibodies were detected by MAT shortly after the first week p.i. and for the following 8–12 weeks [66,67]. Leptospiruria was found in both antibody-positive and -negative cats, supporting the hypothesis that urinary shedding can happen both at an early stage and at a later stage of infection, as reported previously [2]. Leptospiraemia was found in both antibody-positive (Table 3: cat 3, cat 7) and -negative cats (n = 2), and this would suggest that seroconversion can occur after the initial infection when leptospiraemia is still ongoing, as reported in experimental studies [66,67]. The positivity of these cats was at the cut-off level, and a longitudinal evaluation could clarify if leptospiraemia can coexist only with a low antibody titer. No cats were simultaneously positive in blood and urine PCRs. Therefore, according to molecular and antibody assays, four potential infection patterns (Table 4) were identified in the present study, but their interpretation is not easy because little information is available from experimental studies and natural follow-up infections [67]. Interpretation of paired MAT titers collected one or two weeks apart is suggested to confirm a recent infection [2], and a single titer interpretation can limit the MAT sensitivity and specificity, and this could be a limitation of the present study. Other limitations of this study were the lack of culture of biological samples (blood, urine and tissues) as definitive proof of infection [2] and the lack of follow-up to avoid false negative results due to intermittent urine shedding. 

Few studies have investigated possible risk factors for *Leptospira* spp. exposure in cats. These were old age [14,26,29], being an older cat > 1 [30] or ≥4 years old [8], being a cat considered a hunter by the owner, the presence of another cat in the household [19,30], living close to dairy cattle herds [9], being a shelter cat, and having access to the outdoors [31,65] were associated with higher *Leptospira* spp. positivity rates. Moreover, leptospirosis is considered a seasonal disease, and heavy rainfall or flooding were associated with human and animal outbreaks [2]. In a previous study conducted in Quebec, the antibody prevalence among cats was statistically higher between June and August, which are the warmest and most humid months of the year in Quebec [19]. In another study conducted in Iowa, the risk of antibody positivity was significantly higher in spring than in summer or fall [15]. Moreover, in the present study, urinary *Leptospira* DNA shedding was more frequently found in cats enrolled during spring, but no association with age group, lifestyle, origin or environment, as reported in previous studies, was found. It is not easy to explain the spring rise of urinary DNA shedding we observed. Spring rainfall levels are variable in Southern Italy and various factors influence the dynamics of the murine populations, which play an important role in *Leptospira* epidemiology. 

Cats can be infected with leptospires, but clinical signs seem to be rare, and infection is usually clinically inapparent [3]. However, clinical signs have been reported in some infected cats [3]. Previous studies described cases of feline leptospirosis reporting anorexia, lethargy, dehydration, weight loss, polyuria and polydipsia, vomiting, hematuria, uveitis, lameness, ascites and hepatomegaly [23,34]. In the present study, association between *Leptospira* spp. exposure and clinical signs was not found, however positive cats presented with various physical abnormalities such as reduced BCS and muscle mass, enlarged lymph nodes, conjunctivitis and keratoconjunctivitis, stomatitis, nasal discharge, vomiting and diarrhea, alopecia, hyperkeratosis, scaling and abscesses. Some CBC abnormalities, such as neutrophilia [23,25] or neutropenia [34], monocytosis [25], lymphocytosis [34] or lymphopenia and thrombocytopenia [23], were reported in previous case reports of leptospirosis in cats. In the present study, significant associations with anemia, neutrophilia, monocytosis and eosinopenia, compatible with an inflammatory condition and stress response, were found. Significant associations were also found between other inflammation markers, such as hypoalbuminemia and hyperglobulinemia, and *Leptospira* spp. positivity, and a significant association with increased ALT activity in antibody-positive cats was found. The liver is one of the major target organs of leptospires, and in dogs, a mild liver enzyme increase with possible worsening to severe liver failure and signs of hepatic encephalopathy can occur [2]. In cats, liver involvement is rarely reported [27,34], with mild increases in liver enzymes (alkaline phosphatase (ALP), ALT and aspartate aminotransferase (AST). After the end of leptospiraemia, leptospires can persist in sites like the renal tubules with the development of interstitial nephritis [2]. Interstitial nephritis [23,36], azotemia [23,34], low urine specific gravity, proteinuria and ultrasound changes (marked decrease in the definition of the corticomedullary junction, irregular kidney shape) [23] have been reported in cats with *Leptospira* infection. Some studies also investigated the association between *Leptospira* spp. and chronic kidney disease (CKD), but the results are still controversial [19,24,27]. In the present study, possible associations between increased sCr or BUN values, the presence of low USG or proteinuria and *Leptospira* spp. positivity were investigated, but no significant associations were found. However, the lack of a longitudinal evaluation of the *Leptospira* spp.-positive cats cannot exclude the possibility that chronic renal damage could develop later.

## 4. Materials and Methods

### 4.1. Power and Sample Size

Assuming a 3.3% prevalence of leptospiral DNA shedding and 17.9% of anti-*Leptospira* antibodies in cats (17.9%) [27], a sample size of about 110–115 cats was required (95% CI; 4.5% precision for the prevalence of DNA shedding and 7.0% precision for antibody prevalence).

### 4.2. Study Sites, Cat Enrollment and Sampling Procedures

Between January 2018 and May 2019, cats were enrolled in southern Italy at two veterinary clinics located in Sicily (Ospedale Veterinario Universitario Didattico, Università degli Studi di Messina, Messina) and Calabria (Clinica Veterinaria Camagna, Reggio Calabria). Signalment, history and physical examination findings were collected, and the data that were recorded are listed in Table 1 and Table 2 and the supplementary Table S1.

Three to five milliliters of blood were taken from each cat: one milliliter was placed into a K_3_EDTA tube, used within 24 h for a complete blood count (CBC), and the leftovers were stored at −20 °C until DNA extraction. The remaining blood was used to perform blood smears and to obtain serum after clotting in a plain tube and centrifugation. Serum was stored at −20 °C until further use for biochemical and antibody testing. Urine samples (about five ml) were obtained by cystocentesis or free catch and used for urinalysis within two hours after collection. Within 24 h after collection, urine supernatant was used for the evaluation of the urine protein to creatinine ratio (UPC), and aliquots of urine samples were stored at −20 °C for PCR following the preparation described by Sprißler and others [8]. Briefly, urine was centrifuged at 13,000 rpm for 15 min at room temperature within 24 h after collection. The supernatants were discarded and the pellets were washed with phosphate buffered saline (PBS) and transferred into an Eppendorf tube (Eppendorf, Hamburg, Germany). After a second centrifugation step (13.000 rpm, room temperature, 15 min), the supernatant was discarded, and the pellet was resuspended in 180 µL animal tissue lysis (ATL) buffer (Qiagen, Hilden, Germany) and stored at −20 °C until DNA extraction.

### 4.3. Clinicopathological Evaluation

The clinicopathological parameters statistically evaluated are listed in the supplementary Table S1. The CBC was performed using a laser haematology analyzer (IDEXX ProCyteDx^®^ Hematology Analyzer, Idexx Laboratories, Westbrook, ME, USA). Blood smears were stained with May-Grünwald-Giemsa stain and examined to assess the morphology of blood cells, platelet estimate, leukocyte differential count and the detection of haemoparasites [68]. 

The biochemical profile was performed by the Catalyst Dx^®^ Chemistry Analyzer (Idexx Laboratories, Westbrook, ME, USA), liquid chromatography-mass spectrometry for the evaluation of symmetric dimethylarginime (SDMA) (IDEXX Laboratories, Novara, Italia S.r.l) and by a latex agglutination reaction on an automated analyzer AU480 for the evaluation of serum amyloid A (SAA) (Beckman Coulter, Brea, California at the Department of Veterinary Medicine, Cambridge University, UK). 

Urinalysis was performed by dipstick analysis (Combur 9 Test strips, Roche Diagnostics, Indianapolis, Indiana, USA), urine specific gravity (USG) was measured by a Vet 360 refractometer (Reichert, Seefeld, Germany) and microscopic evaluation of urine sediment by using the Kova glasstic slides (Kova International, Garden Grove, CA, USA). The UPC was assessed with the Catalyst Dx^®^ Chemistry Analyzer (Idexx Laboratories, Westbrook, ME, USA).

### 4.4. Microscopic Agglutination Test (MAT)

Anti-*Leptospira* antibodies (Ab) were evaluated by a microscopic agglutination test (MAT), performed at the World Organization for Animal Health (OIE) and National Collaborating Centre for Reference and Research on Leptospirosis, Amsterdam, the Netherlands. MAT was performed following the technique described by Goris and Hartskeerl [69]. Serial two-fold dilutions of serum were tested from 1:20 to 1:640. A positive value was considered when antibody titers were ≥1:20 [8]. Fourteen serovars (Arborea, Ballum, Bratislava, Canicola, Copenhageni, Grippotyphosa, Icterohaemorragiae, Hardjo, Lora, Mini, Patoc, Poi, Pomona, Tarassovi) belonging to 11 serogroups (Australis, Ballum, Canicola, Grippotyphosa, Icterohaemorrhagiae, Javanica, Mini, Pomona, Sejroe, Semaranga, Tarassovi) were used as antigens. The *Leptospira* species, serogroups, serovars and strains evaluated are described in Table 6. Reference strains, from the collection of the OIE and National Collaborating Centre for Reference and Research on Leptospirosis, Amsterdam University Medical Center (Amsterdam, The Netherlands) were used.

### 4.5. DNA Extraction from K3EDTA Blood and Urine Samples

DNA was extracted from 200 µL of K_3_EDTA blood using the PureLink Genomic DNA kit (Invitrogen, Carlsbad, CA, USA) according to the manufacturer’s instructions. At the end of the extraction procedure, DNA was eluted in 100 µL of PureLink genomic elution buffer and stored at −20 °C until used.

DNA was extracted from urine using a Qiagen DNA Micro Extraction kit (Qiagen, Hilden, Germany) according to the tissue manufacturer’s protocol but with the lysis period reduced to 1 h. To elute DNA, 54 µL Qiagen AE buffer was used.

### 4.6. Polymerase Chain Reaction for Detection of Leptospira DNA

DNA extracted from K_3_EDTA blood and urine samples was tested with PCR described by Ahmed and others [70]. Primers and probe sequences targeting *lipL32* gene-specific for pathogenic *Leptospira* (LipgrF2, LipgrR2, and LipgrP1) and the internal set primers, probe, and synthetic internal control template sequences (IntoF2, IntoR2, IntoP1, and PlasintS1) are listed in Table 7. Between 100–500 copies per reaction of genomic DNA extracted from *Leptospira* interrogans strain Kantorowic was used as a positive control. The PCR was performed, including the internal control template to monitor the reaction performance and double-distilled DNase/RNase-Free water as a negative control. All samples, as well as positive control and negative control, were tested in duplicate. Results were considered positive if Ct values were recorded in at least one duplicate and were ≤40.

### 4.7. Statistical Analysis

Descriptive statistic was performed for all the evaluated numerical variables. Chi-squared test or Fisher’s exact test were used to evaluate the relationship between anti-*Leptospira* Ab positivity (Ab^+^), *Leptospira* DNA in urine (uDNA^+^), blood (bDNA^+^), urine and/or blood (DNA^+^), Ab positivity and/or DNA in urine (Ab^+^/uDNA^+^), Ab positivity and/or DNA in urine and/or blood (Ab^+^/DNA^+^) and variables found in all statistical units, in accordance with some of the aforementioned categories, are described in Table 1 and Table 2 and the Supplementary Table S1. Fisher’s exact test was used to evaluate the relationship between the four infection patterns (u^+^ and/or b^+^ and Ab^−^, u^+^ and/or b^+^ and Ab^+^, u^−^ and/or b^−^ and Ab^+^, u^−^ and/or b^−^ and Ab^−^) obtained with molecular (*Leptospira* PCR u/b^−^DNA^+^) and antibody (Ab^+^) tests related to *Leptospira* and the investigated variables are reported in Table 1 and Table 2 and the supplementary Table S1. Fisher’s exact test was also used to evaluate the relationship between the various types of antibody or molecular positivity described above and the type of anemia (regenerative/non regenerative; mild/moderate/severe; micro/normo/macrocytic; hypo/normochromic) and reticulocyte hemoglobin (RETIC-HGB) (low/normal). This statistical analysis was performed using GraphPad Prism Software. 

Spearman’s Rho test was used to measure the strength of correlation between *Leptospira* Ab^+^, uDNA^+^, bDNA^+^, DNA^+^, Ab^+^/uDNA^+^, Ab^+^/ DNA^+^ and variables related to clinical findings, CBC and biochemical profile parameters described in Table 1 and Table 2 and the Supplementary Table S1. Spearman’s Rho test was used to measure the strength of correlation between the four infection patterns obtained with molecular and antibody investigations related to *Leptospira* and the investigated variables reported in Table 1 and Table 2 and the supplementary Table S1. Univariate and multivariate logistic regression analysis models were developed to identify predictive factors for uDNA^+^, bDNA^+^, Ab^+^ of categorical variables sex (males/females), age (junior: 6–24 months; adult: 25–96 months; senior: >96 months), lifestyle (indoor/outdoor/indoor-outdoor), origin (foundling/not foundling), environment (urban/suburban/rural), cohabitation with dogs (yes/no) and enrollment season (spring/ summer, autumn, winter). This statistical analysis was performed using SPSS 22.0 for Windows. *p*-values lower than 0.05 were considered statistically significant.

## 5. Conclusions

In the present study, cats were frequently infected by or exposed to *Leptospira* spp. in southern Italy, and feline infection seems to be caused by the same serovars found in other animal species in Italy. The spring season was the only detected risk factor for urinary DNA shedding. This means that cats can have a role in the epidemiology of leptospirosis, as an additional reservoir or just as sentinels for a risk of infection. Moreover, changes in CBC, ALT and some markers of inflammation found in *Leptospira* spp.-positive cats are potentially compatible with a pathogenic effect. 

## Figures and Tables

**Table 1:** Data from signalment and history of enrolled cats (n (%)) and cats positive for *Leptospira* spp. according to antibody positivity (Ab^+^) and PCR positivity from urine (uDNA^+^) and blood (bDNA^+^).

Variable	All Cats	Ab^+^	uDNA^+^	bDNA^+^
Region				
Sicily	26 (23.2)	4 (23.5)	1 (10.0)	3 (75.0)
Calabria	86 (76.8)	13 (76.5)	9 (90.0)	1 (25.0)
Sex				
Male	51 (45.5)	8 (47.1)	2 (20.0)	2 (50.0)
Female	61 (54.5)	9 (52.9)	8 (80.0)	2 (50.0)
Age group				
Junior (6–24 months)	28 (25.0)	4 (23.5)	3 (30.0)	0
Adult (25–96 months)	61 (54.5)	8 (47.1)	6 (60.0)	3 (75.0)
Senior (>96 months)	23 (20.5)	5 (29.4)	1 (10.0)	1 (25.0)
Lifestyle and Origin				
Outoor	35 (31.2)	4 (23.5)	3 (30.0)	1 (25.0)
Outdoor and indoor	16 (14.3)	4 (23.5)	2 (20.0)	1 (25.0)
Indoor	61 (54.5)	9 (53.0)	5 (50.0)	2 (50.0)
Single-cat household	17 (27.9)	2 (22.2)	2 (40.0)	0
Multi-cat household	29 (47.5)	3 (33.3)	2 (40.0)	0
Rescue cattery	15 (24.6)	4 (44.5)	1 (20.0)	2
Foundling cat	27 (44.3)	4 (44.5)	3 (60.0)	2
Not-foundling cat	34 (55.7)	5 (55.5)	2 (40.0)	0
Cohabitation with dogs	15 (13.4)	2 (11.8)	2 (20.0)	0
No cohabitation with dogs	97 (86.6)	15 (88.2)	8 (80.0)	4
Enrollment Season				
Autumn	15 (13.4)	3 (17.7)	2 (20.0)	1 (25.0)
Winter	61 (54.5)	9 (52.9)	2 (20.0)	3 (75.0)
Spring	33 (29.5)	4 (23.5)	6 (60.0)	0
Summer	3 (2.6)	1 (5.9)	0	0
Environment				
Urban	84 (75.0)	12 (70.6)	8 (80.0)	3 (75.0)
Suburban	26 (23.2)	5 (29.4)	2 (20.0)	1 (25.0)
Rural	2 (1.8)	0	0	0
**Total**	**112**	**17/111**	**10/111**	**4/109**

**Table 2:** Data from clinical findings of enrolled cats (n (%)) and cats positive for *Leptospira* spp. according to antibody positivity (Ab^+^) and PCR positivity from urine (uDNA^+^) and blood (bDNA^+^).

Variable	All Cats	Ab^+^	uDNA^+^	bDNA^+^
Mucous membranes				
Normal	106 (94.6)	17	10	4
Pale	5 (4.5)	0	0	0
Jaundice	1 (0.9)	0	0	0
Body Condition Score < 3/5 ^^^	11 (9.8)	4 (23.5)	1 (10.0)	1 (25.0)
Muscle Condition Score > ¼ ^^^	15 (13.4)	4 (23.5)	0	1 (25.0)
Lymph node enlargement	27 (24.1)	4 (23.5)	3 (30.0)	2 (50.0)
Respiratory tract signs	10 (8.9)	0	1 (10.0)	0
Gastrointestinal signs	4 (3.6)	0	1 (10.0)	0
Skin lesions	14 (12.5)	3 (17.6)	2 (20.0)	1 (25.0)
Ocular lesions	6 (5.4)	2 (11.8)	0	1 (25.0)
Oral lesions	33 (29.5)	4 (23.5)	5 (50.0)	0
**Total**	**112**	**17/111**	**10/111**	**4/109**

^ = body condition and muscle condition scores were rated following a 5/5 [63] and 4/4 [64] scoring system, respectively.

**Table 3:** Description of species, serogroups, serovars, strains found, number of cats for each strain and description of the relative antibody (Ab) titer, cat code (ID), sex, age (months), region of origin (C: Calabria; S: Sicily), month and season of sampling, and negativity (−) or positivity (+) in urine (u^+^) or blood (b^+^) found with PCR.

Species	Serogroup	Serovar	Strain	n (%)	Ab Titer	ID	Sex	Age	Region	Month	Season	u^+^	b^+^
*L. borgpetersenii*	Javanica	Poi	Poi	9 (52.9)	20	7	F	48	S	February	Winter	−	+
					20	2	F	48	C	January	Winter	−	−
					20	8	F	8	S	April	Spring	+	−
					20	9	F	17	C	November	Autumn	+	−
					20	10	F	12	C	January	Winter	−	−
					20	11	M	192	S	January	Winter	−	−
					20	12	M	36	C	January	Winter	−	−
					20	13	M	48	C	January	Winter	−	−
					40	14	F	48	S	April	Spring	−	−
*L. borpetersenii*	Ballum	Arborea	Arborea	3 (17.6)	20	16	F	6	C	January	Winter	+	−
					40	6	M	120	C	April	Spring	+	−
					80	17	F	120	C	January	Winter	−	−
*L. borpetersenii*	Mini	Mini	Sari	1 (5.9)	80	6	M	120	C	April	Spring	+	−
*L. borgpetersenii*	Ballum	Ballum	Mus 127	1 (5.8)	80	1	F	6	C	February	Winter	−	−
*L. interrogans*	Australis	Bratislava	Jez Bratislava	5 (29.4)	20	2	F	48	C	January	Winter	−	−
					20	3	M	168	C	October	Autumn	−	+
					20	4	M	72	C	November	Autumn	−	−
					40	5	M	9	C	July	Summer	−	−
					320	6	M	120	C	April	Spring	+	−
*L. interrogans*	Australis	Lora	Lora	1 (5.9)	80	6	M	120	C	April	Spring	+	−
*L. interrogans*	Pomona	Pomona	Pomona	1 (5.9)	20	15	M	120	C	April	Spring	−	−

**Table 4:** Infection patterns obtained by molecular and antibody assays, with the number of cats showing a specific pattern and their significant associations with the investigated variables compared to the *Leptospira* u and/or b and Ab negative pattern.

*Leptospira* PCR-u/b	Ab	n	Variable	*p*	OR	95% CI
Positive	Positive	6	Monocytosis	0.0206	10.14	1.964–47.64
Positive	Negative	8	Neutrophilia	0.0027	19.14	3.528–106.2
			Albumin decreased	0.0154	11.25	2.213–56.95
			Markers of inflammations	0.0303	∞	1.167–∞
Negative	Positive	11	Anemia	0.0329	5.286	1.441–18.9
			Lymphocytosis	<0.0001	750	41.35–7904
			Eosinopenia	0.0315	7.714	1.614–33.66
Negative	Negative	86	-	-	-	-

u/b = PCR performed in urine and blood samples; a positive result is related to at least one tissue, while a negative result concerns both of them; Ab = result of testing for antibody detection; n = number of cats; markers of inflammation = one or several of the following abnormalities: LAI, increased SAA, increased GLOB, decreased ALB; ∞ = 100% of positive cats showed at least one clinicopathological sign of inflammation; - = no significant associations found. Results of Fisher’s exact test are represented by *p* values, OR (odds ratio) and 95% CI (confidence intervals).

**Table 5:** Statistically significant haematological changes associated with *Leptospira* spp. DNA and antibody positivity.

*Leptospira* Positivity	Anemia		Neutrophilia		Monocytosis		Eosinopenia		Hypoalbuminemia		Hyperglobulinemia		Increased ALT	
	FE	Srho	FE	Srho	FE	Srho	FE	Srho	FE	Srho	FE	Srho	FE	Srho
Ab^+^ *versus* Ab^−^	NS	rs = 0.198 *p* = 0.037	NS	NS	*p* = 0.0001 OR = 24.82 95%CI = 4.973–125.2	rs = 0.283 *p* = 0.004	NS	NS	NS	NS	NS	NS	NS	rs = 0.372 *p* = 0.047
u^+^ *versus* u^−^	NS	NS	NS	NS	NS	rs = 0.203 *p* = 0.038	NS	NS	NS	NS	*p* = 0.0328 OR = 4.803 95%CI = 1.229–16.55	NS	NP	NS
b^+^ *versus* b^−^	NP	NS	NS	rs = 0.200 *p* = 0.042	NS	NS	NP	NS	NS	NS	NS	NS	NS	NS
DNA^+^ *versus* DNA^−^	NS	NS	*p* = 0.0164 OR = 5.571 95%CI = 1.548–22.4	rs = 0.288 *p* = 0.003	NS	rs = 0.208 *p* = 0.036	NS	NS	NS	NS	NS	NS	NS	NS
Ab^+^ and/or u^+^ *versus* Ab^−^ and/or u^−^	*p* = 0.015 OR = 4.32 95%CI = 1.434–12.23	rs = 0.252 *p* = 0.008	*p* = 0.0818 OR = 3.067 95%CI= 0.8726–9.158	rs = 0.218 *p* = 0.027	*p* = 0.0076 OR = 5.357 95%CI = 1.664–17.27	rs = 0.292 *p* = 0.003	*p* = 0.0487 OR = 4.75 95%CI = 1.249–17.35	NS	NS	NS	NS	NS	NP	NS
Ab^+^ and/or DNA^+^ *versus* Ab^−^ and/or DNA^−^	*p* = 0.0399 OR = 3.792 95%CI = 1.282–10.7	rs = 0.228 *p* = 0.016	*p* = 0.0398 OR = 3.773 95%CI = 1.246–10.75	rs = 0.257 *p* = 0.008	*p* = 0.013 OR = 4.625 95%CI = 1.471–14.48	rs = 0.265 *p* = 0.007	NS	NS	*p* = 0.0345 OR = 4.471 95%CI = 1.227–15.98	rs = 0.229 *p* = 0.020	NS	NS	NP	NS

Ab^+^ (antibody positivity), u^+^ (molecular positivity in urine), b^+^ (molecular positivity in blood), DNA^+^ (molecular positivity in urine and/or blood), Ab^+^ and/or u^+^ (antibody and/or molecular positivity in urine), Ab^+^ and/or DNA^+^ (antibody and/or molecular positivity in blood and/or urine). FE = Fisher’s Exact test; Srho = Spearman’s rho test; *p* = *p* value; OR = odds ratio; CI = confidence interval; rs = Spearman’s rank correlation coefficient; NS = not significant; NP = not performed; ALT = alanine aminotransferase.

**Table 6:** *Leptospira* species, serogroups, serovars and strains evaluated.

Species	Serogroup	Serovar	Strain
*L. borgpetersenii*	Ballum	Ballum	Mus 127
*L. interrogans*	Canicola	Canicola	Hond Utrecht IV
*L. interrogans*	Australis	Bratislava	Jez Bratislava
*L. kirschneri*	Grippotyphosa	Grippotyphosa	Duyster
*L. interrogans*	Icterohaemorragiae	Icterohaemorragiae	Kantorowic
*L. borgpetersenii*	Javanica	Poi	Poi
*L. interrogans*	Icterohaemorrhagiae	Copenhageni	M20
*L kirschneri*	Grippotyphosa	Grippotyphosa	Moskva V
*L. interrogans*	Pomona	Pomona	Pomona
*L. interrogans*	Sejroe	Hardjo	Hardjoprajitno
*L. borgpetersenii*	Tarassovi	Tarassovi	Perepelitsin
*L. interrogans*	Australis	Lora	Lora
*L.borpetersenii*	Ballum	Arborea	Arborea
*L.borpetersenii*	Mini	Mini	Sari
*L.biflexa*	Semeranga	Patoc	Patoc I

**Table 7:** List of the sequence of *lipL32*, internal set primers, probe and synthetic internal control used in the study according to *Ahmed* and others [70].

Oligo ID	Sequence	Sequence Source
**LipgrF2**	5′CGCTGAAATGGGAGTTCGTATGATTTCC3′	*lipL32*
**LipgrR2**	5′GGCATTGATTTTTCTTCYGGGGTWGCC3′	*lipL32*
**LipgrP1**	5′FAM GGCGAAATCGGKGARCCAGGCGAYGG3′BHQ1	*lipL32*
**IntoF2**	5′TAGAATCATTGAATCTATCACATCTCATG3′	Internal Control
**IntoR2**	5′TTGAACTAAATGTAGACTAAAGATGATCG’3	Internal Control
**IntoP1**	5′TxRd TTCACATTAACATTCAATAATCAATCATGAA3′BHQ2	Internal Control
**PlasintS1**	5′CTATAGAATCATTGAATCTATCACATCTCATGT ACTTCACATTAACATTCAATAATCAATCATGAATTAATTCAAT TTCTGATATGAATCGATCATCTTTAGTCTACATTTAGTTCAATATATC3′	Internal Control artificial template

## Data Availability

The data set analyzed for the current study is available from the corresponding author upon reasonable request.

## Supplementary Materials

The following are available online at https://www.mdpi.com/article/10.3390/pathogens11101129/s1, Table S1: Data from complete blood count, and biochemical profile of enrolled cats (n (%)) and cats positive for *Leptospira* spp. according to antibody positivity (Ab^+^) and PCR positivity from urine (uDNA^+^) and blood (bDNA^+^).
