# Supplementary material for: Leptospira spp. Prevalence in Cats from Southern Italy with Evaluation of Risk Factors for Exposure and Clinical Findings in Infected Cats
Source: Pathogens. 2022 Sep 30;11(10):1129. doi: 10.3390/pathogens11101129 (PMC9609655; doi:10.3390/pathogens11101129)
Supplement: Supplementary file 1 [file pathogens-11-01129-s001.zip › pathogens-1938740-supplementary-final version.pdf]

Table S1. Data from complete blood count, and biochemical profile of enrolled cats (n (%)) and cats positive for *Leptospira* spp. according to antibody positivity (Ab+) and PCR positivity from urine (uDNA+) and blood (bDNA+).

Variable	All Cats	Ab+	uDNA+	bDNA+
Anemia (Hb < 8.82 g/dL)	15/112 (13.4)	5/17 (29.4)	3/10 (30.0)	0/4
Neutrophilia (>11.32 K/μL)	15/105 (14.3)	3/17 (17.6)	3/8 (37.5)	2/4 (50.0)
Neutropenia (<2.07 K/μL)	5/105 (4.8)	0/17	0/8	0/4
Lymphocytosis (>7.57 K/μL)	2/105 (1.9)	1/17 (5.9)	0/8	0/4
Lymphopenia (<0.83 K/μL)	2/105 (1.9)	0/17	0/8	0/4
Monocytosis (>0.74 K/μL)	14/105 (13.3)	6/17 (35.3)	3/8 (37.5)	1/4 (25.0)
Eosinophilia (>1.73 K/μL)	3/105 (2.9)	1/17 (5.9)	0/8	0/4
Eosinopenia (<0.15 K/μL)	8/105 (7.6)	3/17 (17.6)	1/8 (12.5)	0/4
Basophilia (>0.29 K/μL)	1/102 (1.0)	0/17	0/8	0/4
Thrombocytosis (>660 K/μL)	2/103 (1.9)	0/17	0/8	0/4
Thrombocytopenia (<136 K/μL)	19/103 (18.4)	2/17 (11.8)	2/8 (25.0)	1/4 (25.0)
Increased SDMA (>14 μg/dL)	26/111 (23.4)	3/17 (17.6)	3/10 (30.0)	2/4 (50.0)
Increased sCr (>2.4 mg/dL)	6/112 (5.4)	1/17 (5.9)	1/10 (10.0)	0/4
Increased BUN (>36 mg/dL)	11/108 (10.2)	1/16 (6.3)	1/10 (10.0)	0/4
Decreased BUN (<16 mg/dL)	4/108 (3.7)	1/16 (6.3)	0/10	0/4

Hb = hemoglobin; ˇ= proteinuria was defined when UPC was ≥ 0.2; Hb = hemoglobin; SDMA = symmetric dimethylarginine; sCr = serum creatinine; BUN = blood urea nitrogen; PHOS = phosphorus; USG = urine specific gravity; UPC = urine protein to creatinine ratio; SAA = serum amyloid A; TP = total proteins; ALB = albumin; GLOB = globulins;

ALT = alanine aminotransferase; ALP = alkaline phosphatase; AST = aspartate aminotransferase; GGT = gamma glutamyl transferase; LAI = leukocyte alterations suggestive of inflammation including the presence of neutrophil left shift, toxic neutrophils or reactive lymphocytes; ~markers of inflammation = one or several of the following abnormalities: LAI, increased SAA, increased GLOB, decreased ALB.
